# Implementing a community-based antimicrobial stewardship intervention in Malaysia

**DOI:** 10.1177/17571774241251650

**Published:** 2024-05-07

**Authors:** Ali Haider Mohammed, Angelina Lim, Bassam Abdul Rasool Hassan, Ali Blebil, Juman Dujaili, Dinesh Sangarran Ramachandram, Hawar Sardar Hassan, Arooj Abid

**Affiliations:** 1 School of Pharmacy, 65210 Monash University Malaysia, Bandar Sunway, Malaysia; 2 Department of Pharmacy, 531608 Al-Rafidain University College, Baghdad, Iraq; 3 2541 Faculty of Pharmacy and Pharmaceutical Sciences Monash University, Clayton, VIC, Australia; 4 Murdoch Childrens Research Institute, Royal Children’s Hospital, Parkville, VIC, Australia; 5 Swansea University Medical School, 7759 Swansea University, Swansea, UK; 6 Department of Dentistry, 449486 Komar University of Science and Technology, Kurdistan-Region, Iraq; 7 Department of Radiology, Anwar-Sheikha Medical City, Kurdistan Region, Iraq; 8 Department of Public Health 66692 , Health Services Academy, Islamabad, Pakistan

**Keywords:** Antimicrobial stewardship, community pharmacy practice, antimicrobial resistance education and training, antibiotic resistance prevention, Malaysia healthcare system

## Abstract

**Background:**

In Malaysia, the unregulated use of antibiotics and lack of awareness about antimicrobial resistance (AMR) among pharmacists pose significant challenges. Implementing community-based Antimicrobial Stewardship (AMS) initiatives is crucial to address the rising AMR.

**Methods:**

We developed a bespoke AMS intervention, aligned with the World Health Organization’s AMS modules, as a 2-day online educational seminar for community pharmacists. The effectiveness of the workshop was evaluated using pre- and post-seminar questionnaires, focusing on AMS knowledge and attitudes towards antimicrobial usage.

**Results:**

Among 528 participants, 489 completed both questionnaires. Pre-seminar, only 59% correctly understood the concept of antibiotic resistance reversibility, which improved to 85.9% post-seminar (*p* = .002). The average AMS knowledge score increased from 5/10 to 8/10 post-intervention (*p* < .05). A significant improvement was also noted in pharmacists’ ability to select appropriate antibiotic therapies, particularly for urinary tract infections, with an increase from 78% to 90% correct responses.

**Conclusion:**

The AMS seminar was well-received and significantly improved the AMS knowledge of community pharmacists. The results underline the need for more AMS-focused interventions in this demographic in Malaysia, contributing to the development of formalized AMS programs. Such initiatives are expected to enhance antibiotic use awareness, encourage optimal antibiotic practices, and positively shift professional conduct in community settings.

## Introduction

Antimicrobial resistance (AMR) has surfaced as a critical issue challenging healthcare systems globally in the 21st century (1). AMR occurs when microorganisms adapt, developing resistance to medications, leading to infections that become difficult to treat and increasing the risk of spreading these resistant organisms, often called “superbugs,” within communities (Rochford et al., 2018). This resistance has contributed to increased death rates, longer hospital stays, and higher healthcare costs due to infections caused by bacteria that resist multiple drugs (Dadgostar, 2019; Rochford et al., 2018). A 2019 global report estimated that about 5 million individuals contracted serious infections from antibiotic-resistant bacteria, resulting in at least 1.27 million deaths annually (Morrison and Zembower, 2020; Rochford et al., 2018). With antibiotics widely available and frequently used, resistance has been escalating, especially in developing countries, where misuse of these drugs is common (Boolchandani, D’Souza and Dantas, 2019). As resistance grows, first-choice antibiotics lose effectiveness, requiring more toxic and expensive alternative treatments (Getahun et al., 2020). In Malaysia, a significant 16% rise in annual antimicrobial usage was recorded between 2009 and 2010. Moreover, patients needing systemic antibiotic therapy typically face the highest healthcare costs (Hofer, 2019). Hence, there is a pressing need for more effective antibiotic use strategies (Hofer, 2019).

Antimicrobial Stewardship programs are pivotal in countering AMR and curbing antibiotic overuse. AMS involves the careful and responsible management of antimicrobial medications (Hofer, 2019). The key components of AMS include monitoring antibiotic use, educating healthcare providers and patients, implementing targeted interventions, and establishing leadership roles to ensure accountability in antibiotic prescribing and usage (Akpan et al., 2020; Nathwani et al., 2019).

In Malaysia, antibiotics are supposed to be prescribed by doctors or dispensed by community pharmacists with a valid prescription (Bo et al., 2022). However, the verification of appropriate antibiotic use is often inadequate, and there have been instances of community pharmacists dispensing antibiotics without a prescription. This highlights an urgent need for AMS initiatives within the community pharmacy setting in Malaysia, where there is a lack of interventional AMS studies and minimal literature on AMS knowledge among community pharmacists (Erku, 2016). While AMS programs have been effectively introduced in other countries (Saha et al., 2019), this research aims to assess the impact of an online, live AMS educational workshop, based on WHO recommendations (Akpan et al., 2020; World Health Organization, 2019a), on the AMS understanding and antibiotic selection practices of community pharmacists.

## Methods

### Study design and study participants

The research was structured as a pre- and post-intervention study, centering on an online AMS educational workshop conducted over 2 days in November 2022 as the intervention. Community pharmacists across Malaysia were invited to participate through emails and announcements posted in social media groups tailored for community pharmacists on platforms like Facebook® and Telegram®. Eligible participants were those i) registered with the Pharmacy Board of Malaysia and ii) actively employed (part-time or full-time) in a community pharmacy setting. Interns, recent graduates, and those unwilling to engage in the study were excluded. The efficacy of the intervention was assessed via pre- and post-workshop questionnaires. Invitations for the workshop were disseminated starting from October 1, 2022, with a closing date for registrations on November 5, 2022. The workshop itself was conducted over 2 days, on November 12 and 13, 2022. This timeline from the invitation to the workshop attendance ensures adequate time for pharmacists to register and prepare for the event. The sample size be calculated based on the number of community pharmacists in the population, which is 3892 (Erku, 2016). According to the Raosoft Sample Size Calculator, using a population size of 3892 community pharmacists and a response distribution of 50%, these parameters were chosen to ensure a robust and reliable sampling strategy, balancing accuracy with feasibility. Based on these inputs, the Raosoft calculator recommended a minimum sample size of 350 participants. This size is deemed sufficient to confidently represent the views and practices of the community pharmacist population in Malaysia while maintaining a high standard of statistical validity.

### Pre- and Post-questionnaire development

The pre- and post-questionnaire was approximately 10 min long and consisted of multiple-choice and short answer items, and it was adapted using previous AMS knowledge questionnaires (Erku, 2016; Sarwar et al., 2018; Tahoon et al., 2020). The pre- and post-questionnaire was the same, with the exception that the post-questionnaire also requested an evaluation of the workshop itself and satisfaction with the intervention. It is pertinent to note that the content validation was undertaken by three practicing clinical pharmacists, each possessing over a decade of experience in AMS and infectious diseases. Their extensive experience ensured the relevance and accuracy of the questionnaire. The pilot testing was put through a Cronbach’s alpha reliability test, which returned a value of 0.878, indicating that it is reliable and has good internal consistency. There are four distinct parts to the English-language questionnaire. The first part of the questionnaire included demographics (gender, age, level of education, years of experience, and description of place of practice). The second part focused on testing AMR and AMS knowledge using case scenarios. The third part measured pharmacists’ attitude towards AMS using a 5-point Likert scale (5: strongly agree, 4: agree, 3: neutral, 2: disagree, and 1: strongly disagree). Lastly, the fourth component measured confidence in determining the most appropriate antibiotic medication using four simulated cases from a community pharmacy (viral conjunctivitis, cellulitis, tooth infection, and urinary tract infection). Each response deemed most appropriate to a knowledge question resulted in a one-point score for the participant; no deductions were given to errors/inappropriate/inappropriate suggestions. The total knowledge score was then converted to a scale from 0 to 10. The pharmacists were given the pre-workshop questionnaire using an online link 20 min before the start of the workshop; if the form hadn’t been completed by then, the link would have been disabled. At the end of the workshop, the same group of participants was given the same survey link as the post-questionnaire.

### Educational workshop

The AMS online, live, educational workshop took place over the course of two sessions on Saturday, November 12, and Sunday, November 13, 2022. Zoom was the virtual platform used to host the meetings. PowerPoint presentations, films, and clinical case scenarios were delivered during the course of each session’s 90 min. The workshop content was developed with input from the WHO’s online AMS training modules (Stewardship WA, 2020). Two clinical pharmacists with over 10 years of expertise in the field of infectious diseases reviewed the authors’ work and provided feedback before it was published. The educational workshop was delivered by a team of four expert presenters, comprising two clinical pharmacists specialized in infectious diseases, a microbiologist, and an AMS program coordinator. Each presenter brought unique insights and expertise, enriching the workshop’s content and delivery. Theoretical topics including (1) antibiotic fundamentals and therapeutic concepts, (2) AMR, (3) AMS, and (4) the pharmacist’s involvement in stewardship programs were covered in four separate presentations. During the first session of the course, pharmacists discussed a booklet containing infection guidelines, including those for respiratory infections, skin infections, and urinary tract infections. The pre-workshop questionnaire was provided to participants 20 min prior to the commencement of the workshop, with a stipulation that it must be completed before the link was disabled. Conversely, the post-workshop questionnaire was distributed immediately after the workshop’s conclusion, with participants allotted 30 min to complete it. This precise timing ensures a focused and prompt assessment of the workshop’s impact. The participation in this workshop was free of charge, and there was no incentive to be given for participation. The participation in this study was completely voluntary, and if any of them decided not to participate there would not be any negative consequences. Participants were also informed that they may stop participating at any time (before or during the start of answering the questionnaire), and if they did so, their responses would not be counted.

### Data analysis

**Figure 1.**
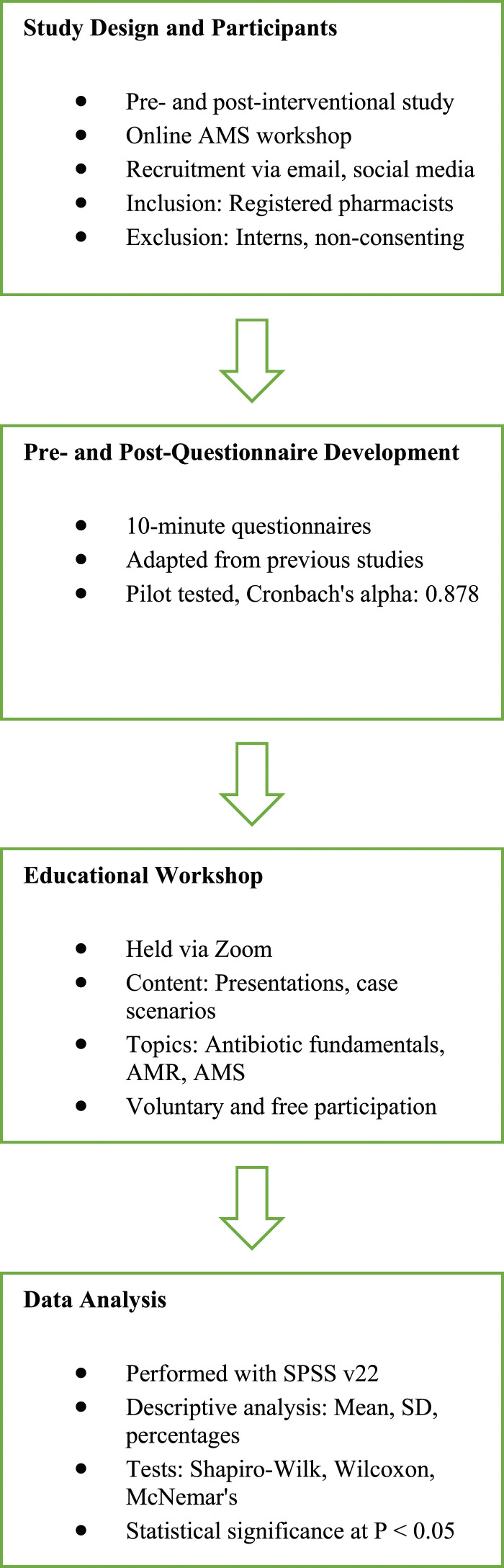
Flowchart representing the study approach.

The analysis of the data was performed with SPSS version 22. During the descriptive analysis, researchers used measures like mean and SD for the continuous variables and percentages for the qualitative ones. Shapiro–Wilk test (where *p* > .05 indicates normally distributed continuous variable) was used to check for normality. The pre-workshop and post-workshop differences in continuous variables were analyzed using the Wilcoxon sign rank test, while the differences in categorical variables were analyzed using McNemar’s test. Values of Cronbach’s over 0.7 suggest sufficient internal consistency in the questionnaire (Association GAotWM, 2014). All tests were two-tailed, and a *p*-value of less than 0.05 was considered statistically significant as shown in Figure 1.

## Results

### Socio-demographics

Five hundred twenty community pharmacists attended the training. However, 489 of them filled out the questionnaire completely before and after the workshop (a completion rate of 94%). The average age of the study participants was 32.6 years (SD 7.5), the vast majority were male (*n* = 359, 73.4%), and 84.8% had at least a baccalaureate degree in pharmacy (*n* = 415). The majority of participants (*n* = 340, 69.5%) worked in private, independent pharmacies, while the remainder 30.5% (*n* = 149) are employed by national or regional pharmacy chains. Almost 31% of the participants (*n* = 139) said they have attended an AMS-related session in the past. Table 1 displays the detailed sociodemographic data.

**Table 1.** Demographic characteristics of the study sample (*N* = 489).

Demographic	Median (IQR)	*n* (%)
Age (years)	32.6 (7.5)	
Gender		
• Female		130 (26.6)
• Male		359 (73.4)
Educational level		
• BPharm/pharmD		415 (84.8)
• Clinical Master of Pharmacy		30 (6.1)
• PhD		44 (9.1)
Community practice experience (years)	7.6 (5.2)	
Site of work		
• Independent community pharmacy^a^		340 (69.5)
• Chain community pharmacy^b^		149 (30.5)
Place of residence		
• Urban		310 (63.3)
• Non-urban		179 (36.7)
Have you ever attended a course/workshop on AMS programmes?		
• Yes		139 (28.5)
• No		350 (71.5)

^a^Independent community pharmacy is a single store with a sole proprietor or may consist of several stores owned by an individual or small group.

^b^Chain community pharmacy is an organization or individual set-up/open more than four medical stores or pharmacies.

### Awareness about AMS

**Figure 2.**
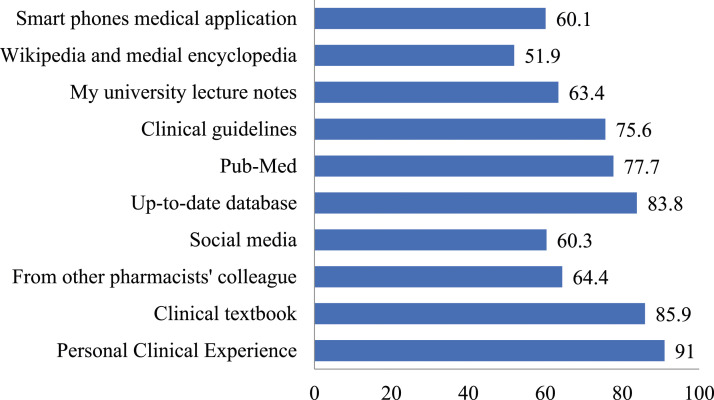
Community pharmacist’s main source of knowledge about antimicrobial and antimicrobial use during their practice (*n* = 489).

Participants were polled on where they learnt about antibiotics and proper antibiotic usage (Figure 2). The majority of participants (*n* = 445, 91%) reported using either clinical textbooks (*n* = 420, 85.9%) or an up-to-date database (*n* = 410, 83.8%). In terms of what resources participants use, the majority used PubMed (*n* = 380, 76.7%) and clinical guidelines including Malaysian National Antimicrobial Guideline (NAG) and WHO guidelines (*n* = 370, 75.6%), respectively. At the same time, half of the participants relied on the Internet or social media for research purposes, with sources including coworkers (*n* = 315, 64.40%), university lecture notes (*n* = 289, 63.4%), and social media (*n* = 295, 49.0%).

**Figure 3.**
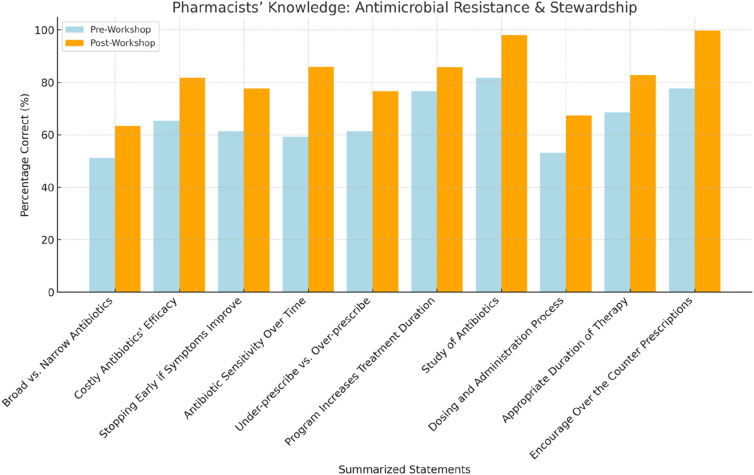
Community pharmacists knowledge towards antimicrobial resistance.

Part two of the pre- and post-questionnaire findings based on AMS knowledge is detailed in Table 2. Overall, most pharmacists reported learning new information from the workshop. During pre-surveys, only 77.7% (*n* = 380) of respondents most appropriately identified the falsehood of the statement “purpose of AMS is to encourage over the counter prescription of antibacterial agents”; however post-surveys, this number had increased to 99.7% (*n* = 488) (*p* = .043). The median participant knowledge score increased from 7 (IQR = 5) before the session to 8 (IQR = 6) after the workshop (*p* = .003). Table 2 and Figure 3 provide further information.

**Table 2.** Community pharmacists’ knowledge about antimicrobial resistance and AMS (*N* = 489).

Statements	Most appropriate *n* (%) pre-workshop	Most appropriate *n* (%) post-workshop	*p*-Value^a^
Broad-spectrum antibiotics should always be used in place of narrow-spectrum antibiotics to reduce resistance^b^	250 (51.1)	310 (63.3)	0.001^c^
The efficacy of the more expensive antibiotic is associated with better efficacy and lower resistance^b^	320 (65.4)	400 (81.7)	0.001^c^
If symptoms improve before the full course of antimicrobial is completed, your patient can stop taking it^b^	300 (61.3)	380 (77.7)	0.005
Antibiotic with resistance problem can become sensitive over time^b^	290 (59.3)	420 (85.9)	0.002
It is always better to under-prescribe antibiotics than over-prescribe^b^	300 (61.3)	375 (76.6)	0.002
AMS is a program that increases the treatment duration to ensure therapeutic efficacy^b^	375 (76.6)	420 (85.8)	<0.001^c^
AMS is a study of antibiotics^d^	400 (81.7)	480 (98.1)	0.015
AMS is a process that involves a suitable antibiotics dosing and route of administration^d^	260 (53.1)	330 (67.4)	0.016
AMS is a process that involves the appropriate duration of antibiotics therapy^d^	335 (68.5)	405 (82.8)	0.019^c^
The role of AMS is to encourage over-the-counter prescription of antibiotics agents^b^	380 (77.7)	488 (99.7)	0.043
Knowledge score (out of 10), median (IQR)	5 (3)	8 (6)	0.003^c,e^

^a^McNemar’s test.

^b^False.

^c^Significant at 0.05 significant level.

^d^True.

^e^Using Wilcoxon signed-rank test.

### Pharmacists’ ability to appropriately select most appropriate antibiotic therapy for virtual community pharmacy cases

**Figure 4.**
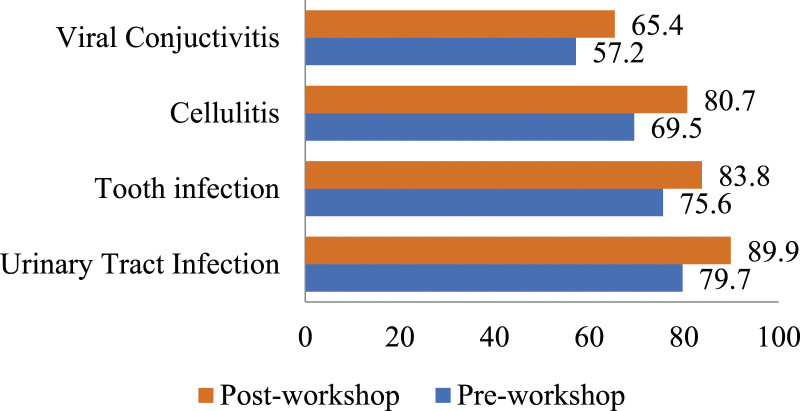
Selection of correct empirical therapy pre- and post-workshop for simulated cases.

Figure 4 elucidates the outcomes from the fourth section of the questionnaire, which focused on empirical therapy preferences across four clinical scenarios. The data reveals a notable enhancement in participants’ aptitude for selecting accurate antibiotic therapy for urinary tract infections (UTIs). Prior to the workshop, 79.7% (*n* = 390) of participants most appropriately identified the appropriate therapy, a figure which escalated to 89.9% (*n* = 440) post-workshop, a statistically significant increase (*p* = .003). Similarly, for bacterial sinusitis, initial proficiency was observed at 57.2% (*n* = 280) among pharmacists in most appropriately determining the non-necessity of antibiotics for viral conjunctivitis. This percentage improved to 65.4% (*n* = 320) following the workshop, marking a statistically significant enhancement (*p* = .048). Notably, pharmacists also demonstrated statistically significant improvements in their responses to the other two clinical situations—dental infections and cellulitis—after participating in the training (*p* < .05).

In summary, Figure 4 highlights the significant improvement in the ability of community pharmacists to appropriately select the most appropriate antibiotic therapy for various community-based clinical cases, both before and after attending the educational workshop. The improvements were statistically significant across all cases: UTI (*p* = .003), tooth infection (*p* = .025), cellulitis (*p* = .001), and viral conjunctivitis (*p* = .048).

**Figure 5.**
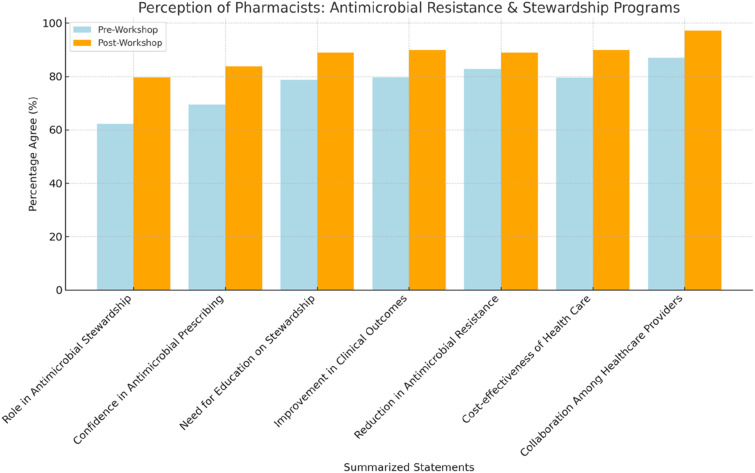
Perception of community pharmacists towards AMR and stewardship programs.

There were seven statements used to evaluate pharmacists’ attitudes towards the AMS program; most respondents had a very positive attitude towards the programs both before and after the workshop, with significant changes in their responses after the workshop (P0.05 for all statements). Sixty-three percent of participants (*n* = 305) agreed that they should play a pivotal role in antibiotic stewardship and infection prevention. After attending the workshop, this figure rose to 79.7% (*n* = 390). In addition, 69.5% of participants (*n* = 340) agreed/strongly agreed that community pharmacists should have appropriate knowledge on AMS, and this number was increased to 83.8% (*n* = 410) after the workshop, showing that community pharmacists understand the significance of the stewardship program (Table 3; Figure 5).

**Table 3.** Perception of community pharmacists towards antimicrobial resistance and the importance of AMS programs (*N* = 489).

	Pre-workshop	Post-workshop	
Statements	Strongly Agree/Agree *n* (%)		*p*-Value^a^
Community pharmacists have a responsibility to take a prominent role in AMS and infection prevention	305 (62.3)	390 (79.7)	0.019
I feel confident about my knowledge and practice in the area of antimicrobial prescribing	340 (69.5)	410 (83.8)	0.014
Sufficient education on AMS should be given to community pharmacists	385 (78.7)	435 (88.9)	0.045
AMS will improve the patient’s clinical outcomes	390 (79.7)	440 (89.9)	0.007
AMS will reduce antimicrobial resistance	405 (82.8)	435 (88.9)	0.003
AMS improves the cost-effectiveness of healthcare sectors	389 (79.5)	440 (89.9)	0.035
AMS improves the collaboration between healthcare providers	425 (86.9)	475 (97.1)	0.019

^a^McNemar’s test.

In a nutshell, the educational initiative appears to have significantly bolstered the community pharmacists’ awareness and understanding of antimicrobial resistance and stewardship, as evidenced by the comparative analysis of pre- and post-workshop knowledge assessments. Notably, there was an appreciable elevation in most appropriate responses post-intervention, with the acknowledgment of broad-spectrum antibiotics usage to mitigate resistance increasing from 51.1% to 63.3%, and the recognition of AMS’s role in enhancing therapeutic efficacy advancing from 76.6% to 85.8%. Additionally, the pharmacists’ perception of their pivotal role in AMS and infection prevention was markedly reinforced, rising from 62.3% in agreement pre-workshop to 79.7% post-workshop. The overwhelming satisfaction with the workshop’s content and delivery underscores the program’s effectiveness, corroborated by near-universal agreement on its educational value. These findings reflect a substantive “inflation”—a marked increase—in the population’s awareness and competence regarding AMS, indicative of the initiative’s profound impact.

### Satisfaction with the AMS workshop

**Figure 6.**
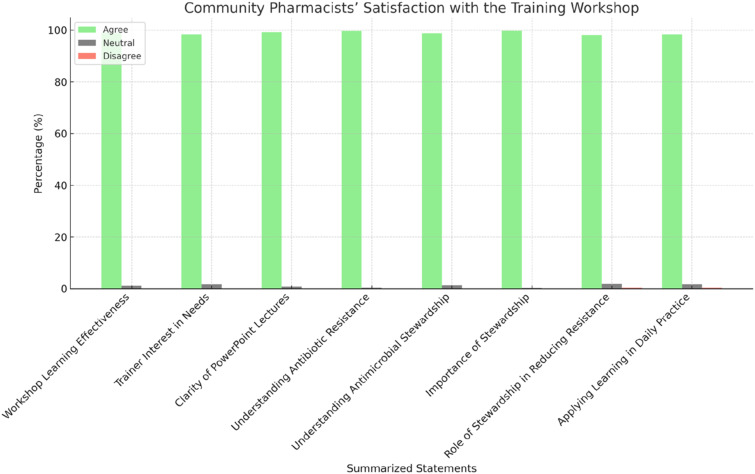
Community pharmacist’s satisfaction with the educational intervention.

As an additional part of the post-workshop questionnaire, each participant rated their level of satisfaction with the workshop based on eight statements (Table 4; Figure 6). Participants reported gaining a deeper understanding of the principles of antimicrobial stewardship through the workshop. (*n* = 487, 99.6%) and that they learned new information (*n* = 484, 98.9%) about antimicrobial resistance (*n* = 480, 98.1%). Overwhelmingly, participants in the educational session agreed or strongly agreed that the knowledge gained about the significance of stewardship programs is anticipated to influence participants’ daily professional practices. (*n* = 488, 99.7%).

**Table 4.** Community pharmacists’ satisfaction with the training workshop (*N* = 489).

Statements	Strongly Agree/Agree *n* (%)	Neutral *n* (%)	Strongly Disagree/Disagree *n* (%)
The online workshop helped me to learn	484 (98.9)	5 (1.1)	0 (0.0)
Trainers showed an interest in my needs during this workshop	481 (98.3)	8 (1.7)	0 (0.0)
The PowerPoint lectures were easy to follow and understand	485 (99.1)	4 (0.8)	0 (0.0)
The workshop made me understand the concept of antibiotic resistance	487 (99.6)	2 (0.4)	0 (0.0)
The workshop made me understand the concept of AMS	483 (98.7)	6 (1.3)	0 (0.0)
The educational workshop helps me understand the importance of antimicrobial stewardship	488 (99.7)	1 (0.3)	0 (0.0)
The cases integrated into the workshop gave me clear examples of the role of AMS in reducing antimicrobial resistance	480 (98.1)	7 (1.89)	2 (0.4)
The educational workshop will allow me to practice what I had learned in my daily practice at my practice site	481 (98.3)	6 (1.69)	2 (0.4)

## Discussion

The global escalation in mortality and morbidity due to communicable and infectious diseases is a pressing concern, with notable increases observed in various countries (Rochford et al., 2018). Malaysia, in particular, grapples with challenges linked to the accessibility of antibiotics and the absence of AMS protocols in community settings. The World Health Organization has emphasized the importance of training health workers in antimicrobial resistance (AMR) through its curricula guide (World Health Organization, 2019a). Additionally, several studies have underscored the urgency of augmenting AMS awareness in such environments (Akpan et al., 2020; Majumder et al., 2020; Nathwani et al., 2019; Saha et al., 2021). In 2014, Malaysia made a significant step by adopting the Protocol on AMS Program in Healthcare Facilities, thereby formalizing AMS in its healthcare system. This protocol delineates the objectives of an AMS team and the roles of its members, emphasizing the pivotal role of pharmacists (Lai et al., 2022). Despite this formal adoption, the practical implementation of these principles in Malaysia remains limited. Our study is pioneering in evaluating the effectiveness of a community-based AMS intervention in Malaysia.

The results of our study are promising, indicating that community-based AMS interventions can indeed be effective. There was a significant enhancement in AMS knowledge and attitudes post-intervention. The participants showed a considerable improvement in selecting the most appropriate antibiotic therapy for various community pharmacy scenarios (*p* = .05). The initial knowledge about AMS was relatively low, with only a third of participants being familiar with the concept before the intervention. This lack of awareness is consistent with findings in countries like Egypt and Ethiopia, where less than half of medical practitioners were acquainted with AMS (Saleh et al., 2021). However, countries like South Africa and Australia have reported higher awareness levels (Bishop et al., 2018). The limited exposure of Malaysian community pharmacists to AMS and AMR education is a contributing factor to this knowledge gap. Interestingly, participants in our study, despite their limited awareness of AMS, demonstrated substantial baseline knowledge of AMR, paralleling findings from similar research in Egypt and Ethiopia (Tahoon et al., 2020).

Participants exhibited a positive perception of AMS both before and after the intervention. This aligns with the findings of Satterfield et al. (2020), which indicated a moderate understanding of AMS’s role among participants prior to an educational program (Satterfield et al., 2020). However, there was a notable deficiency in knowledge regarding the pharmacist’s active role in AMS. The educational workshop conducted in our study proved effective in enhancing understanding of both empirical and directed therapy, which are key aspects of WHO’s AMS principles (Falcone et al., 2019; World Health Organization, 2019b). The high participation rate and positive feedback from Malaysian pharmacists reflect a strong inclination towards improving practices in this area. The majority found the workshop exceedingly beneficial, underscoring the value of tailoring programs to suit the specific expertise and practice needs of pharmacists (May et al., 2021).

Our study boasts a significant strength in its large sample size and high response rate (92.6%), ensuring comprehensive representation of community pharmacies across Malaysia. This widespread participation enhances the potential for widespread dissemination of AMS principles throughout the country. While the workshop was conducted in English, which is the predominant language of practice among Malaysian pharmacists, it inadvertently excluded non-English-speaking individuals. The voluntary nature of the workshop may have also introduced a selection bias, attracting pharmacists who are more proactive in seeking knowledge and improvement. Ongoing research is directed at evaluating the long-term impact of this intervention on everyday practices.

### Importance of the study

Our study significantly improved the knowledge of community pharmacists in Malaysia about how to use antibiotics most appropriately. This improvement could lead to better choices in dispensing medicines and help reduce the misuse of antibiotics. Over time, the study could have a big impact on public health in Malaysia, especially in fighting the problem of bacteria becoming resistant to antibiotics. By showing how useful these educational workshops are, our study supports the idea of having more training programs like this. These changes can lead to healthier patients, lower medical costs, and a change in how people think about using antibiotics. The study not only helps pharmacists but can also guide future training for other healthcare workers. In the end, our study is an important step in reducing the problems caused by the misuse of antibiotics in Malaysia, highlighting the need for ongoing education and awareness in healthcare. To extend and sustain the educational achievements of our AMS workshop, we propose several strategies. Firstly, the incorporation of ongoing training sessions, possibly semi-annually or annually, would reinforce the core concepts of AMS and introduce new developments in the field. Additionally, integrating AMS topics into continuous professional development programs for pharmacists can ensure a steady engagement with the subject. Furthermore, leveraging digital platforms for e-learning modules can provide flexible and accessible learning opportunities. These initiatives are crucial for maintaining momentum and ensuring that the knowledge and practices imparted during the initial workshop are not only retained but also evolved in line with the latest in antimicrobial stewardship. As a recommendation for future study, the finding that 20% of workshop participants did not recognize their role in AMS warrants further exploration. Understanding the underlying reasons for this perspective is crucial for devising targeted interventions. This could be approached through qualitative methods like follow-up interviews or focused group discussions to delve into the pharmacists’ reservations or misconceptions about their role in AMS. Subsequently, tailored strategies, such as awareness campaigns emphasizing the critical role pharmacists play in AMS, sharing success stories and best practices from peers, and providing specific resources and support for AMS implementation, could be developed. These efforts aim to cultivate a more uniform and comprehensive understanding of the pivotal role community pharmacists play in antimicrobial stewardship, ultimately fostering a more engaged and proactive approach across the profession.

## Conclusion

The AMS educational workshop conducted in Malaysia was notably successful, effectively increasing AMS knowledge among community pharmacists. The findings underscore the need for more targeted AMS interventions in Malaysia, aimed at designing official AMS programs at the community level. Such initiatives are crucial for raising awareness, optimizing antibiotic use, and, most importantly, facilitating tangible changes in daily practice.

## Data Availability

Data and other materials are available upon request from the corresponding authors.
